# Care of Children’s Teeth

**Published:** 1889-11

**Authors:** J. R. Callahan


### Care of Children's Teeth.

BY J. R. CALLAHAN, D.D.S.

Read before the Seventh Ohio District Dental Society, Sept. 17, 1889.

A short time since a circumstance came to my notice that attracted my attention. A wealthy gentleman and lady of this city were having their teeth taken care of by one of the best and most experienced dentists of the city, while their family of three or four children were having a like service performed by one of the cheapest, as well as one of the most incompetent, dentists in this State. If I could mention names you all would recognize a family who would not hesitate a moment on a matter of expense for any of the members of the household.

Under these circumstances I could think of but two reasons that would account for the above circumstance: 1st. The expert dentist may have had little or no patience with the children, and was harsh and rough with them; 2nd, the parents have been allowed to think that the children's teeth are of no consequence, and so long as they keep the children from crying with "toothache" they are doing all that is necessary for them.

There are many operators who are not qualified in any way to take charge of children under any circumstances, least of *all* to fill their teeth. Lack of sympathy for the child, or failure to appreciate the positiou the child occupies, leads to as much trouble as a lack of ability to properly treat the case from a professional standpoint. Some try to deceive the little ones; a thing, I think, very wrong. They soon make up their minds as to your disposition toward them. You may make a great fuss over them; ask their names; tease them about the baby; offer them candy as a reward for good conduct; give them your refuse rubber dam, and other such nice things, but if your heart is not in what you say and do, they will detect your insincerity at once, and they will not be slow to let you know the verdict. Very seldom will deceit be of even temporary advantage; never a permanent good.

I have known dentists to dceive the parents by pleasant manners, etc., and then handle the child with a great deal of impatience. I saw a well-known dentist filling a tooth for a little girl. While the mother was present he was a *model* of patience and gentleness. Presently the lady left the operating room. The manner of the dentist changed at once; he was there simply for business; then he went at that tooth in a manner that soon had the little girl crying and wanting to get out of the chair. He pushed her back into the chair, at the same time saying, "Sit down, you little fool, or I'll box your ears."

A brother student once told me that he gave them a little taffy till he conld get the mouth open and the instrument in place, and the child completely at his mercy, *then*, said he, "I give them H—." Such men are brutal, and should not attempt to do dental work for those who are not old enough to protect themselves. They do irreparable injury. No one can fill teeth well while in a bad temper, and the result is the patient loses confidence in dentistry. They grow up with a dread of the dental chair that they can not overcome. It is well known to all of you that it requires the greatest skill and best judgment to treat children's teeth properly. A very small per cent. of the profession are adapted, in all ways, for this kind of practice. It would be far better if the dentist who feels that he does not have the necessary patience, or time for the children, to send them to some one specially adapted to that kind of practice.

We owe a duty to the parents also, viz.: they should be instructed, not only in regard to their own teeth, but those of the children. The development and care of teeth in early life should be impressed upon their minds in as forcible a manner as possible. Not only to watch them closely, but to obtain the advice and co-operation of a *reliable* dentist.

The fact that intelligent people, who are willing to do any thing in the world for the good of their children, allow them to go to the most inferior dentist, while they themselves go to the very best, shows plainly that the *best* dentist is *sadly* neglecting his duty as a teacher. He certainly has not edeavored to instruct the parents on any dental subject. A little knowledge of the mouth and teeth would drive any thoughtful parent to carefully consider the condition of their children's mouths, and the dentist would be rewarded by the everlasting gratitude of the child and parent.

---

SENILE MICROBIA-MANIA.—The *Revue de Therapeutic* says: A savant of Naples, Dr. Malinconico, has made a greater discovery than the famous elixir of youth of Brown-Sequard. The journals announce very seriously that Dr. Malinconico is about to discover the *microbe of old age.*

The microbe is transmitted, according to the Italian savant, by inheritance, invades with age the entire human organism, ravages and destroys it, producing old age, and finally death.

Dr. Malinconico hopes that he will be able to discover the means to combat, and finally to destroy, this terrible microbe, which will prevent men growing old. The savants are invaluable.—*Times and Register.*

